# *Galleria mellonella* Infection Model Demonstrates High Lethality of ST69 and ST127 Uropathogenic *E. coli*

**DOI:** 10.1371/journal.pone.0101547

**Published:** 2014-07-25

**Authors:** Majed F. Alghoribi, Tarek M. Gibreel, Andrew R. Dodgson, Scott A. Beatson, Mathew Upton

**Affiliations:** 1 Microbiology and Virology Unit, School of Medicine, University of Manchester, Manchester, United Kingdom; 2 King Abdullah International Medical Research Center, King Saud bin Abdulaziz University for Health Sciences, Riyadh, Saudi Arabia; 3 Central Manchester Foundation Trust, Manchester, United Kingdom; 4 Australian Infectious Disease Centre, School of Chemistry & Molecular Biosciences, University of Queensland, Queensland, Australia; 5 School of Biomedical and Healthcare Science, Plymouth University Peninsula Schools of Medicine and Dentistry, Plymouth, United Kingdom; Indian Institute of Science, India

## Abstract

*Galleria mellonella* larvae are an alternative *in vivo* model for investigating bacterial pathogenicity. Here, we examined the pathogenicity of 71 isolates from five leading uropathogenic *E. coli* (UPEC) lineages using *G. mellonella* larvae. Larvae were challenged with a range of inoculum doses to determine the 50% lethal dose (LD_50_) and for analysis of survival outcome using Kaplan-Meier plots. Virulence was correlated with carriage of a panel of 29 virulence factors (VF). Larvae inoculated with ST69 and ST127 isolates (10^4^ colony-forming units/larvae) showed significantly higher mortality rates than those infected with ST73, ST95 and ST131 isolates, killing 50% of the larvae within 24 hours. Interestingly, ST131 isolates were the least virulent. We observed that ST127 isolates are significantly associated with a higher VF-score than isolates of all other STs tested (*P*≤0.0001), including ST69 (*P<0.02*), but one ST127 isolate (strain EC18) was avirulent. Comparative genomic analyses with virulent ST127 strains revealed an IS1 mediated deletion in the O-antigen cluster in strain EC18, which is likely to explain the lack of virulence in the larvae infection model. Virulence in the larvae was not correlated with serotype or phylogenetic group. This study illustrates that *G. mellonella* are an excellent tool for investigation of the virulence of UPEC strains. The findings also support our suggestion that the incidence of ST127 strains should be monitored, as these isolates have not yet been widely reported, but they clearly have a pathogenic potential greater than that of more widely recognised clones, including ST73, ST95 or ST131.

## Introduction

*Escherichia coli* is the major cause of extraintestinal infections including urinary tract infection (UTI), Gram-negative bacteraemia and neonatal meningitis. Uropathogenic *E. coli* (UPEC) are the most frequent cause of UTI, being responsible for up to 85% of community acquired UTI and 40% of nosocomial UTI [1]–[3].

Multilocus sequence typing (MLST) is the current method used to investigate the genetic differences between isolates of UPEC. This method has been used to good effect to identify UPEC as well as other important pathogenic *E. coli* [1],[4]–[6]. Our own work and that of others has highlighted the importance of several leading lineages of UPEC (e.g. Sequence Type 69 (ST69), ST73, ST95, ST127 and ST131) and we have recently suggested that ST127 is a newly evolved clone, with particularly high virulence potential [4]. Numerous other recent studies have highlighted the virulence and antimicrobial resistance of members of these clones [6]–[10].

Based on PCR surveillance of virulence factors, UPEC have been shown to possess multiple virulence-associated determinants that include diverse adhesins, toxins, siderophores, capsule variants and other miscellaneous traits [11]–[14]. Although a great deal of research effort has been devoted to understanding UPEC virulence mechanisms, much remains for further investigation and animal models of UTI are resource intensive.

The larvae of the wax moth *Galleria mellonella* have been used as an infection model to describe and evaluate microbial pathogenicity for a number of bacterial pathogens, including enteropathogenic *E. coli* (EPEC) [15]–[19]. The virulence mechanisms of many pathogens in *G. mellonella* show a high degree of similarity to mammals, including humans [20],[21]. Previous studies have shown a strong and positive correlation of virulence of different pathogens between mouse infection systems and *G. mellonella* [22]–[24]. In this study, *G. mellonella* larvae were used as an *in vivo* model to investigate the virulence of the major lineages of UPEC.

## Material And Methods

### Bacterial Strains

A total of 71 non-duplicate isolates of *E. coli* from patients with UTI were examined in this study. The patients included those presenting in the community and nosocomial infections. All isolates were recovered at clinical bacteriology laboratories at Central Manchester Foundation Trust, Preston Royal Hospital and the Mid Yorkshire Hospital Trust, Wakefield, between 2007 and 2011. The MLST and virulence typing of 57 of these isolates has been previously described [4]. The isolates were selected on the basis of assignment to the major lineages of UPEC, as determined by using the Achtmann MLST scheme, and were from ST69 (n = 11), ST73 (n = 20), ST95 (n = 10), ST127 (n = 10) and ST131 (n = 20), using previous methods [5]. PCR based detection of 29 uropathogen associated virulence factors (previously defined by Johnson et al., 2000 [12]) was carried out for each of the examined isolates.

### Phylogenetic Grouping

Phylogenetic grouping was determined by triplex PCR reaction targeting three DNA markers (*chuA, yjaA* and TSPE4.C2), as described previously by Clermont and colleagues [25].

### Serotyping

Molecular serotyping was performed on all the isolates using a multiplex PCR method to detect 14 *Escherichia coli* serogroups associated with UTI (O1, O2, O4, O6, O7, O8, O15, O16, O18, O21, O22, O25, O75 and O83), as described previously [26]. Isolates that were not able to be typed using this method (i.e. gave negative results with all primer pairs) were classified as nontypable (nt).

### Identification Of Ld50 In *G. Mellonella* Larvae Infection

Larvae of the Greater Wax Moth, *G. mellonella* (GM) were obtained from Live Foods Ltd (Rooks Bridge, UK). Larvae were stored in the dark and used within 10 days of receipt. Larvae were selected to be 15–25 mm in length, having a cream colour with minimal speckling and no grey markings. To prepare UPEC inoculum, strains were grown in nutrient broth overnight at 37°C and collected by centrifugation at 13,000×*g* for two minutes. The cell suspensions were normalised using optical density (OD_600_) and the colony forming units (cfu/ml) were confirmed by viable count assay.

A minimum of three biological replicates of 10 larvae were injected per serial dilution of UPEC (10^2^, 10^3^, 10^4^, 10^5^, 10^6^ and 10^7^ cfu/10 µl) using a Hamilton syringe (26S gauge, 50 µl capacity). Larvae were then incubated at 37°C in the dark and the dilution that killed 50% of the larvae (LD_50_) for each replicate was determined after 24 hours. Larvae were monitored for an additional 120 hours and survival outcome was determined; larvae were considered dead when no response was observed following touch. In addition, 10 larvae were injected with non-pathogenic *E. coli* DH5α to evaluate whether *G. mellonella* larvae were killed by non-infection related reactions and a control inoculation (n = 10) was performed with 10 µl of PBS to measure any lethal effects due to physical injury. An additional control group (n = 10) had no manipulation.

Survival analysis and statistical significance were determined using the log-rank test and the Kaplan–Meier survival curves were plotted using SPSS v.20. The LD_50_ was calculated using probit regression model implemented in SPSS v.20 at a significance level of *P* = 0.05.

### Assessment Of Virulence Of Upec Strains *In Vivo*

Having identified the LD_50_ for each isolate, we investigated the virulence of each strain over 120 hrs to assess the utility of the model for comparison of virulence of the UPEC strains. Larvae were inoculated with a dose corresponding to the LD_50_ and survival followed over 120 hrs, as described above.

### Correlation Analyses

In order to investigate correlations between the LD_50_ and virulence profiles of the isolates from the five STs, data were examined by using the Kruskal-Wallis test, followed by pairwise analysis of differences performing Mann–Whitney U-tests in Prism v.6 (www.graphpad.com/).

The LD_50_ was taken to be a continuous variable and the values for all isolates were used to divide them into three groups; low LD_50_ (∼10^2^–10^3^ cfu/10 µl, the volume initially used to inoculate the larvae), medium LD_50_ (∼10^4^–10^5^ cfu/10 µl) and high LD_50_ (∼10^5^–10^7^ cfu/10 µl). Virulence factors were correlated with LD_50_ using Prism v.6 by fitting Pearson's correlation coefficients between the three LD_50_ groups and virulence factors. The correlation was used to describe the virulence factors that are associated with each group.

### Genome Sequencing And Annotation Of *E. Coli* Ec18 And Ec41

Genomic DNA of ST127 strains EC18 and EC41 (virulent and virulent in GM larvae, respectively) was sequenced using Illumina MiSeq by the Centre for Genomic Research, University of Liverpool. Velvet 1.2.10 software [27] was used to assemble sequence reads of both genomes into contigs. For each strain (EC18 and EC41), a total of 6,666,026 and 4,165,821 sequence reads were assembled into 149 and 178 contigs greater than 200 bp in length with and average depth of coverage of 196.71 and 114.92, respectively. Contigs were ordered according to the complete genome of UPEC ST127 strain 536 (Accession ref |NC_008253; [28]) and annotated using Prokka 1.7 (Prokka: Prokaryotic Genome Annotation System - http://vicbioinformatics.com/) [29]. The Illumina sequence reads are available under the Bioproject PRJEB6308/ERP005824 (Accession Numbers: EC18, ERS497039; EC4, ERS497040). Comparison and visualization of UPEC genomes were carried out using BLAST [30], Artemis comparison tool (ACT) [31], BLAST Ring Image Generator (BRIG) [32], Easyfig [33] and Tablet [34].

### Gap Region Amplification And Sequencing Of The O-Antigen Deletion Region In Ec18

Primers were design to target the gap of the O-antigen deletion region in EC18 strains (gap-F 5′-TCA AGC ACC GAA TAA CCT -3′) and (gap-R 5′-TAC CTG AAG TAC GTA GCC-3′). The primers were designed based on the sequence of the two contigs adjacent to the O-antigen deletion region, for which no direct linkage information was available from the genome sequence. As the size of the expected product was not immediately clear from the genome assembly alone, long-range PCR was performed using Q5 High-Fidelity 2X Master Mix (New England Biolabs). PCR was performed as follows; 98°C for 30 sec, 30 cycles of 98°C for 10 sec, 52°C for 30 sec and 72°C for 30 sec, and a final extension at 72°C for 2 min. Electrophoresis in a 1% agarose gel was used to determine the size of the PCR product by running with Quick-Load 1 kb Extend DNA Ladder (New England Biolabs). The DNA sequence of the amplified product was determined by Sanger sequencing at the Plymouth University Systems Biology Centre using an Applied Biosystems 3130 Genetic Analyzer. Sequence data were assembled using CLC Main Workbench v.6.9.1.

## Results

### Phylogenetic Grouping

Phylogenetic analysis of the 71 strains examined indicated that they belonged to groups B2 (51%), D (48%) and B1 (1%). All ST69 strains belonged to phylogenetic group D, ST73 to both group D (13 strains) and B2 (7 strains), ST95 to both group D (4 strains) and B2 (6 strains), ST127 belonged to groups D (4 strains) and B2 (6 strains), and ST131 belonged to groups D (2 strains), B2 (17 strains) and B1 (1 strain) (Table S1).

### Serotyping

Serotyping of the 71 strains indicated that they belonged to several serogroups with O6 being the most prevalent (32%) followed by O25 (25%), O15 (13%), O2 (7%), O16 (4%), O18 (3%), O22 (1%); 14% were classified as nt (Table S1). Serogroup O6 was seen for all of the ST127 strains, except EC18, which was nt. ST73 (9 strains) and ST95 (5 strains) were also O6. Fifteen strains of ST131 belonged to O25, with the remainder (3 strains) belonging to O16. O25 was also observed for ST73 (2 strains) and ST69 (1 strain). Most of the ST69 strains (n = 6) and 3 ST95 strains were O15. Nontypable strains were observed for all STs (Table S1).

### Upec From St69 And St127 Are Significantly More Pathogenic Towards *G. Mellonella* Than Those From Other Sts

**Figure 1:**
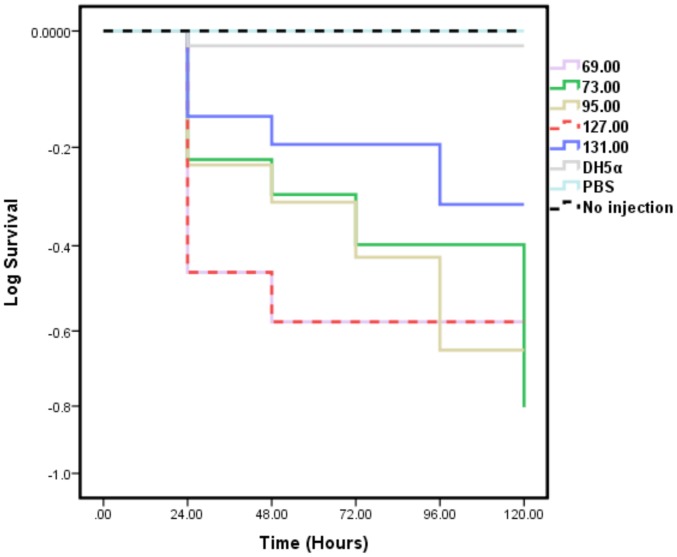
Kaplan-Meier survival analysis of *G. mellonella* larvae following injection of UPEC cells (2.87×10^6^ cfu/ml) of different sequence types (ST). Data presented are the mean of three independent assays with each UPEC isolate. Low larvae mortality was recorded following injection of DH5α. Non-injected larvae and PBS injected larvae showed no mortality. Survival outcome of larvae injected with ST69 and ST127 isolates recorded the highest mortality compared to ST73 (*P*≤0.248), ST95 (*P*≤0.303) and ST131 (*P*≤0.054).

The larvae were injected with a range of inoculum doses to determine the mortality rates of each isolate. An example of the Kaplan-Meier survival analysis for 2.87×10^4^ cfu/10 µl of a strain from each ST investigated is shown in Figure 1. The survival outcome of the tested UPEC clones varied, where ST69 and ST127 showed high mortality rates compared to ST73 (*P*≤0.248), ST95 (*P*≤0.303) and ST131 (*P*≤0.054). Isolates of ST131 were observed to be the least virulent in this model. Larvae injected with non-pathogenic *E. coli* DH5α, showed no sign of stress, with an insignificant level of larval death recorded, indicating that larval death is related to overt pathogenic mechanisms in the UPEC strains. However, an interesting result to emerge from the data was the observation that one of the ST127 isolates (strain EC18) did not show any lethal effects, even for high inoculum doses (up to 2.33×10^7^ cfu/10 µl).

**Figure 2:**
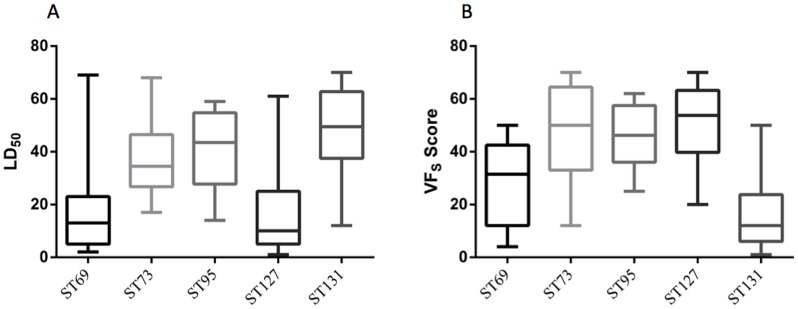
Correlation analysis between the five UPEC sequences types: (A) Low LD_50_ shows significant lethal effects with larvae inoculated with ST69 and ST127 strains. (B) ST127 shows high virulence capacity compared to that of other STs and ST131 shows relatively low virulence capacity.

Lethality tests conducted to investigate LD_50_ confirmed that isolates of ST69 and ST127 were significantly more virulent than those of the other STs tested (Table 1). The median LD_50_ for ST69 and ST127 isolates was 1.59×10^4^ cfu/10 µl (*P*≤0.0021) and 1.17×10^4^ cfu/10 µl (*P*≤0.047), respectively (Figure 2A). Isolate EC18 was not included in statistical calculations, as it had no recordable LD_50_ value.

**Table 1:** Statistical analysis indicates the significant difference in carriage of virulence factors (based on a PCR survey) and lethal effects (Low LD_50_) with larvae inoculated with ST69 and ST127 strains.

STs	ST69		ST127	
	VF (*P* value)	LD50 (*P* value)	VF (*P* value)	LD50 (*P* value)
ST69	-	-	0.02	0.401
ST73	0.0124	0.0023	0.6985	0.0024
ST95	0.0185	0.0079	0.3653	0.0051
ST127	0.02	0.401	-	-
ST131	0.0115	0.0021	0.0001	0.0004

### Deletion Of O-Antigen Gene Cluster In Upec Strain Ec18 Results In Avirulence In *G. Mellonella*

**Figure 3:**
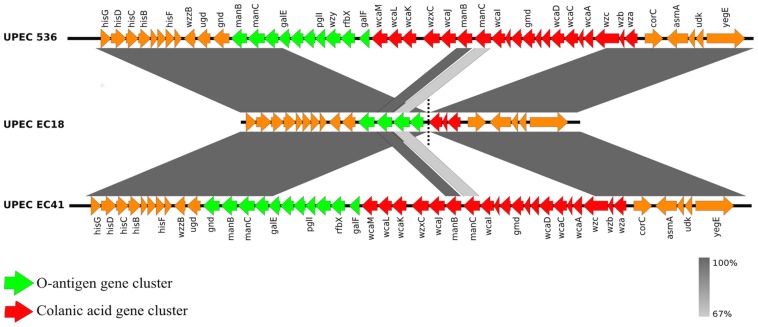
BLASTn comparison of O-antigen gene cluster (green) and colanic acid gene cluster (red) from UPEC ST127 strains 536, EC18 and EC41. The gray shading indicates high nucleotide identity between the sequences (99–100%). In EC18, there is an insertion-sequence (IS1) mediated deletion of most of the O-antigen gene cluster and the colanic acid gene cluster (vertical dotted line denotes contig boundaries in EC18). Figure was prepared using Easyfig [33].

Comparative genomic analysis was carried between strains EC18, EC41 and 536, the only completely sequenced ST127 strain available in the public databases [28]. This analysis indicated an insertion-sequence (IS1) mediated deletion (from *glaE* to *wcaA*) within the O-antigen gene cluster in strain EC18. UPEC 536 is a model UPEC strain used for studies on ExPEC pathogenesis and belongs to serogroup O6 [8]. The O-antigen gene cluster, which is involved in the synthesis of the O-antigen, is encoded by the constituent genes *galF* to *gnd* [35]–[37]. In EC18, there is a deletion of the majority of the O-antigen gene cluster, which results in a contig break in the assembly and sequence evidence of an insertion sequence (IS1) at the position where the deletion occurs (Figure 3). In contrast the O-antigen region was completely assembled into a single contig in the EC41 assembly (Figure 3). To ensure that the missing EC18 O-antigen genes were not encoded elsewhere in the EC18 assembly we carried out a whole genome comparison between the 536 complete genome and the EC18 and EC41 draft genomes (data not shown). Furthermore, BLASTn and BLASTp comparisons with 95 O-antigen sequences, as described by DebRoy and colleagues [36], showed that none of the known O-antigen regions were present in the EC18 assembly. Due to the short insert size of Illumina paired-end sequence data it was not immediately clear from the draft genome assembly of EC18 if there was a single IS1 sequence, or if two or more IS1 along with intervening sequence were arranged in tandem within the O-antigen region. PCR and subsequent Sanger end-sequencing of the product confirmed the presence of a single IS1 sequence replacing the O-antigen region in EC18 from *galE* to the 3′ end of *wzc*, inclusive (Fig 3). The IS1 sequence in EC18 is 100% identical to an IS1 annotated in *Salmonella enterica* subsp. *enterica* serovar Typhimurium str. T000240 (dbj|AP011957). The closest match in the ISfinder database is 96% nucleotide identity to IS1X2 from *E. vulneris* ATCC29943 (gb|Z11605).

**Figure 4:**
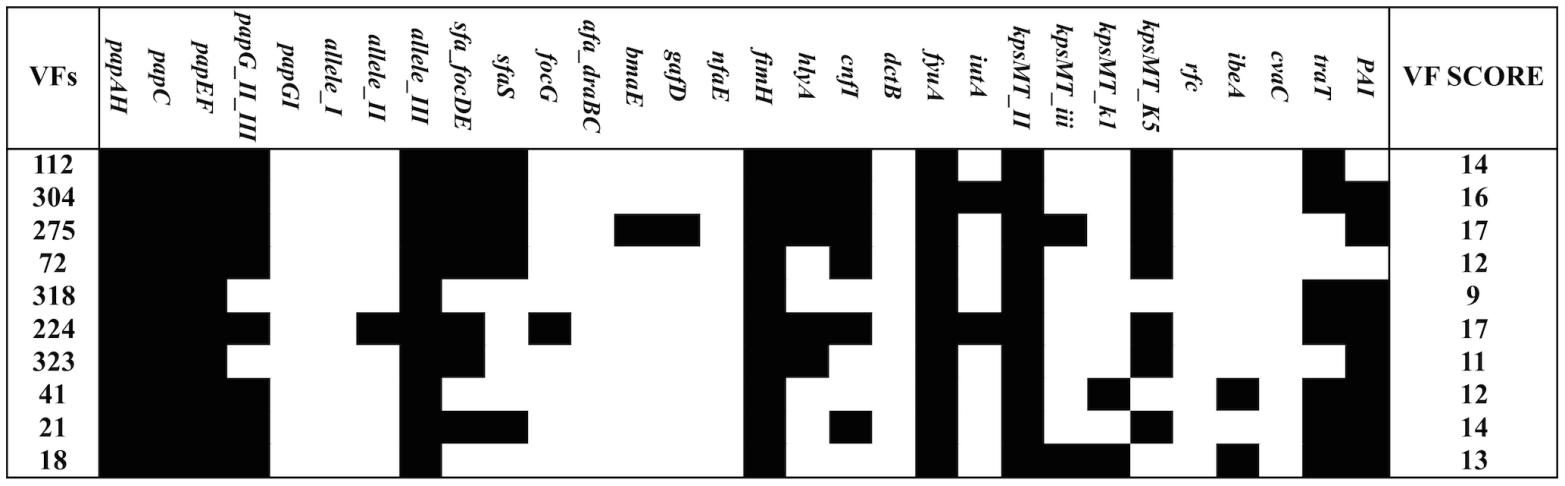
Virulence profile of ST127 strains based on PCR detection of 29 uropathogen associated virulence factors. EC18 (avirulent strain) shows a similar profile compared to other ST127 virulent strains. Black blocks represent positive PCR results and strain numbers are in the left hand column.

Comparison of the virulence genes carried by ST127 strains, using a PCR survey, indicated no other missing gene targets that could clearly explain the difference in virulence between EC18 and other strains (Fig. 4).

### Lethality In The *G. Mellonella* Model Is Correlated With Carriage Of Specific Virulence Determinants

The virulence profile of the UPEC strains examined here was determined by PCR based surveillance of 29 virulence factors (Table 2), which indicated that ST127 isolates are significantly associated with a higher VF-score (the sum of positive PCR test results; Figure 2B) than isolates of the other STs tested (ST73, ST95 & ST131, *P*≤0.0001; ST69, *P*<0.02) (Table 1). All isolates tested were positive for the fragment of the *fimH* gene sought using PCR, so data are not presented in Table 2.

**Table 2:** Prevalence of various UPEC associated virulence factors in the strains studied.

		Prevalence (%) of VF by sequence type					
Category	Specific VF	Total no.	ST69	ST73	ST95	ST127	ST131
		71	(n = 11)	(n = 20)	(n = 10)	(n = 10)	(n = 20)
Adhesion	*papA*	41 (58)	8 (73)	13 (65)	9 (90)	10 (100)	1 (5)
	*papC*	41 (58)	7 (64)	14 (70)	10 (100)	10 (100)	0
	*papEF*	40 (56)	9 (82)	13 (65)	7 (70)	10 (100)	1 (5)
	*papGII,III*	42 (59)	7 (64)	16 (80)	10 (100)	8 (80)	1 (5)
	*allele-II*	27 (38)	6 (55)	11 (55)	9 (90)	1 (10)	0
	*allele-III*	15 (21)	0	4 (20)	0	10 (100)	1 (5)
	*sfa/foc DE*	20 (28)	0	13 (65)	0	7 (70)	0
	*afa/draBC*	10 (14)	0	0	0	0	10 (50)
	*sfaS*	7 (10)	0	2 (10)	0	5 (50)	0
	*focG*	12 (17)	0	10 (50)	1 (10)	1 (10)	0
	*bmaE*	2 (3)	1 (9)	0	0	1 (10)	0
	*gafD*	2 (3)	1 (9)	0	0	1 (10)	0
Toxins	*hlyA*	21 (30)	0	15 (75)	0	5 (50)	1 (5)
	*cnf1*	19 (27)	0	12 (60)	0	6 (60)	1 (5)
	*cdtB*	5 (7)	0	4 (20)	1 (10)	0	0
Siderophore	*fyuA*	69 (97)	10 (91)	19 (95)	10 (100)	10 (100)	20 (100)
	*iutA*	49 (69)	8 (73)	16 (80)	5 (50)	2 (20)	18 (90)
Capsule	*kpsM II*	49 (69)	6 (55)	12 (60)	10 (100)	10 (100)	11 (55)
	*kpsM III*	6 (8)	3 (27)	1 (5)	0	2 (20)	0
	K1	16 (25)	2 (18)	5 (25)	8 (80)	1 (10)	0
	K5	31 (44)	4 (36)	6 (30)	2 (20)	8 (80)	11 (55)
Miscellaneous	*cvaC*	6 (8)	1 (9)	0	5 (50)	0	0
	*ibeA*	7 (10)	0	2 (10)	0	2 (20)	3 (15)
	*traT*	54 (76)	11 (100)	12 (60)	9 (90)	7 (70)	15 (75)
	*PAI*	53 (75)	2 (18)	18 (90)	7 (70)	8 (80)	18 (90)

This supports our previous reports for ST127 strains [4] and the data presented here corroborate the suggested low virulence capacity of ST131 isolates [38],[39].

In order to assess the correlation between the LD_50_ and carriage of specific virulence factors, the continuous variable of LD_50_ was divided into three groups: low, medium and high. The medium and the high LD_50_ groups varied, in terms of constituent STs, but the low LD_50_ (i.e. high virulence) group contained only isolates belonging to ST69 and ST127 (Table S1). It should be stated that not all members of these STs exhibited the same LD_50_ values: in ST69 there were isolates of high (n = 1), medium (n = 5) and low (n = 5) LD_50_; in ST127 these values were 2 (including EC18), 5 and 3, respectively.

Importantly, a low LD_50_ was seen to have a significant positive association with several virulence factors; *papAH, papC, papEF, sfaS, bmaE, gafD* and *kpsMTIII* (Table 3). In contrast, the pathogenicity island marker gene (*PAI*) was negatively associated with a low LD_50_.

**Table 3:** Association of LD_50_ with selected virulence factor.

	Number of isolates	Number of isolates carrying selected virulence factors							
LD_50_ category		*papAH*	*papC*	*papEF*	*sfaS*	*bmaE*	*gafD*	*kpsMTIII*	*PAI*
Low LD_50_	8	7	7	7	3	2	2	3	3
* P* value		0.0436	0.0436	0.0355	0.0001	<0.0001	<0.0001	0.0036	0.0019
Medium LD_50_	51	32	32	30	3	0	0	2	40
* P* value		—	—	—	0.0101	0.0269	0.0269	0.0379	
High LD_50_	12	2	2	3	1	0	0	1	10
* P* value		0.0013	0.0013	0.016	—	—	—	—	—

Underlining indicates a negative association.

Isolates of ST69 were not significantly associated with carriage of a high number of VFs (Figure 2B). The mechanism underlying the pronounced virulence of ST69 isolates in this model has not yet been determined. Previous work has correlated growth rate with lethality in *G. mellonella*, where mortality was associated with proliferation of *Burkholderia* species, *Klebsiella pneumoniae*, *Staphylococcus aureus* or *Streptococcus pneumoniae* within the larvae [19],[21],[40],[41]. However, we investigated growth rate in LB medium and the ST69 isolates we have examined here were in fact seen to have the slowest growth rate, or longest doubling time (data not shown).

## Discussion

UPEC are a major cause of UTI and the severity of the infection is due to the contribution of many virulence factors including adhesins, toxins, siderophores and capsule. The diversity of the virulence factors enable UPEC to escape host immune responses and persist to cause infection [13],[14]. In the current study, *G. mellonella* larvae were used as an *in vivo* model to investigate the virulence of UPEC from the leading lineages known to cause UTI. Previous studies indicate that the *G. mellonella* model is a powerful tool to investigate the virulence of a range of bacterial and fungal pathogens [40],[42],[43]. Of most relevance to the current study is the work of Leuko and colleagues, who demonstrated that pathogenicity of EPEC could be dissected using *G. mellonella* larvae, and that *E. coli* K12 was non-pathogenic [18]. The innate immune systems of insects such as *G. mellonella* display a high degree of similarity to the mammalian immune systems, which make *G. mellonella* an attractive alternative to animal models for investigation of pathogenicity [19],[42],[44],[45]. Plasmatocytes and granulocytes have been identified in *G. mellonella* as types of haemocytes that are involved in phagocytosis, encapsulation and nodule formation, which are important elements in the defence against pathogenic bacteria [46], as suggested for EPEC [18]. In addition, larvae of *G. mellonella* are large enough to allow easy handling, inexpensive to purchase and, being invertebrates, investigations do not require ethical permission.

The findings presented here illustrate different levels of virulence among isolates from the leading UPEC lineages and reveal that these phenotypes are largely conserved within the tested clones. We observed a significant association between lethality and carriage of specific virulence factors, but not with growth rate *in vitro*.

Based on virulence factor surveillance, ST127 has the highest virulence potential, which is consistent with our previous findings [4]. The median LD_50_ of virulent ST127 strains was 1.17 x 10^4^ cfu, almost one log higher than that recently reported for a single strain of EPEC [18]. A previous study by Johnson and colleagues showed that ST127 causes extraintestinal infections in humans, dogs, and cats [8]. The clonal group ST127 includes the reference strain 536, which is a model organism of extraintestinal *E. coli* infections and the first ST127 complete genome to be reported [8],[28]. Other than strain 536, members of ST127 have not been widely reported, presumably because it is a recently evolved clone. Due to its pathogenic potential, ST127 may represent a significant health problem in the future, especially if strains were to acquire extensive antimicrobial resistance.

Comparative genomic analyses were carried out by Hochhut and colleagues between 536 and another reference strain, CFT073, which revealed at least five pathogenicity islands (PAI I-V536) specific to strain 536 [28],[47],[48]. Strain 536 (O6:K15:H31) is well-characterized and it has been demonstrated that it produces various types of fimbrial adhesins, such as S fimbriae (*sfa*) and type 1 and P-related fimbriae [49]. The P-related fimbriae genes and S fimbrial adhesins are located on PAI I536 and PAI II536, respectively and deletion mutants in these regions show decreased potency *in vivo* [49],[50]. These observations are supported by our correlation analysis between LD50 and virulence profiles, which showed that ST127 isolates with significant lethal effects in *G. mellonella* are associated with the fimbrial adhesins *bmaE* (M fimbriae), *gafD* (G fimbriae) *papAH, papC, papEF* (P fimbriae) and *sfaS* (S fimbriae).

It has been demonstrated that EPEC with a defective type III secretion system (T3SS) have reduced virulence in *G. mellonella* [18]. The same paper describes how activation of the Cpx envelope stress response pathway, removing all significant cell envelope associated virulence factors, including T3SS and the bundle forming pillus, will render EPEC avirulent. The data we present here regarding the VFs selected for analysis by PCR, indicate that additional virulence factors contribute to pathogenicity of UPEC in the *G. mellonella* model, but support suggestions that this insect is a valid tool for investigation of the pathogenicity of *E. coli*. We also suggest that, as the ST127 strain 536 is a recognized human pathogen, our observations support the use of *G. mellonella* as a model to indicate potential for causing disease in mammals.

The discovery of a single avirulent ST127 strain, EC18, has allowed a deeper investigation of the mechanisms contributing to survival, a prerequisite for virulence, of *E. coli* in the larvae. All other strains examined had some degree of lethality and the observation of a single avirulent strain in ST127 was rather striking. Comparative genomic analysis between EC18, EC41 and strain 536 revealed a deletion of the O-antigen and the colanic acid gene cluster in EC18. The O-antigen, part of lipopolysaccharide (LPS) present in the outer membrane of Gram-negative bacteria, is a major virulence factor of UPEC. Previous studies have demonstrated that bacterial LPS is important for virulence in the nematode model of infection [52]–[54]. In one study, it was demonstrated that *Salmonella* Typhimurium required an intact LPS to resist the immune response, persist and multiply within *G. mellonella* [52]. A recent study by Browning and others (2013) showed the essential nature of O-antigen production as a key virulence determinant mediating killing of *Caenorhabditis elegans* worms by *E. coli* [53]. Browning demonstrated that regeneration of the O-antigen biosynthesis cluster renders *E. coli* K-12 strain MG1655 pathogenic in *C. elegans*. In this study we demonstrate the importance of the O-antigen gene cluster in the ability of UPEC strain EC18 to kill *G. mellonella*. Given that the VF score for the avirulent ST127 strain EC18 was similar to that for the virulent ST127 strains (Fig. 4), it is clear that loss of O-antigen can supersede the virulence potential of UPEC in *G. mellonella*. The normal bactericidal effects of the innate immune system of higher animals, including *G. mellonella*, play a crucial protective role during bacterial infection [55]–[57]. Antimicrobial peptides in the *G. mellonella* hemolymph are key factors in the humoral immune response against invading microorganisms [58],[59]. Several antimicrobial peptides that are effective against Gram-negative bacteria, including apolipophorin III (apoLp-III), lysozyme and anionic peptide 2, have been identified in the *G. mellonella* hemolymph [60],[61]. It has been suggested that O-antigens and colanic acid provide an effective protective barrier against desiccation, phagocytosis and serum complement-mediated killing, including the action of antimicrobial peptides [62]–[66]. A recent study by Phan and others demonstrated the importance of the O antigen and colanic acid to serum resistance in ST131 UPEC strain EC958 [67]. The study identified 56 serum resistance genes, of which the majority encode membrane proteins or factors involved in LPS biosynthesis. In addition, another study by Sarkar and colleagues showed the important role of O-antigen in the virulence of UPEC were it was demonstrated that the O6 antigen has a major impact on the colonization of the mouse urinary tract [51]. In our study, serotype O6 was the most prevalent (32%) among the 71 examined isolates, which were distributed across different STs in strains that demonstrated different levels of lethality. This indicates that, although the O6 antigen may be important for bacterial survival during UTI, it is not correlated with virulence in the *G. mellonella* hemolymph. Our results suggest that the absence of a functional O antigen and colanic acid gene cluster in EC18 renders the bacteria sensitive to the activity of *G. mellonella* hemolymph. However, the mechanisms that lead to the pronounced lethality of some strains from the ST127 lineage are yet to be deciphered. The genome sequence data we have generated, in combination with publically available sequences for ST69 strains will allow us to begin to explore this in greater depth. We have also demonstrated a correlation between carriage of certain virulence factors with low LD_50_, which warrants further experiments with mutant strains, and their complemented derivatives, to investigate the role of individual virulence factors in pathogenicity in the *G. mellonella* model.

The ST69 lineage is part of phylogenetic group D and is also described as clonal group A (CGA), which has been identified as an important cause of UTI, with CGA-D-ST69 strains being responsible for up to 50% of infections caused by trimethoprim-sulfamethoxazole-resistant isolates [68]–[70]. Analysis of the virulence profile of CGA strains has indicated similarity to O15:K52:H1 isolates, which were found to be more virulent than other *E. coli* [71],[72]. The O15:K52:H1 clonal group is considered to be a widely disseminated and important UPEC lineage [73]. In the current study, ST69 isolates were highly lethal in *G. mellonella*. This may be associated with specific adhesins in this clone, including the *pap* alleles. Interrogation of the VF data recorded during this study did not reveal any obvious similarity in VF profile between the low LD_50_ isolates from ST69 and ST127.

In conclusion, this is the first study to investigate the virulence of UPEC using *G. mellonella* as an *in vivo* model. The findings demonstrate that ST69 and ST127 isolates are, with one exception, highly virulent. We have demonstrated that the O-antigen cluster is essential for resistance to the action of the innate immune response in *G. mellonella*. Given previous studies with ST69 (CGA) and ST127 (strain 536) UPEC, and our demonstration of the correlation between lethality and specific virulence factors, we suggest that the *G. mellonella* model is a good model to study virulence of UPEC strains, and is a useful tool for discovery of candidate vaccine targets. The high virulence potential and lethality of ST127 isolates emphasises the need to perform a comprehensive analysis of the genetics underlying the virulence of members of this clonal group and suggests that increased surveillance for the clone is justified.

## Supporting Information

Table S1 Serotyping, Phylo-grouping, median lethal value LD_50_ and virulence factors of each strain examined from the five UPEC lineages. (XLSX)
